# The Passing Shadow

**Published:** 1903-03

**Authors:** 


### THE PASSING SHADOW.

The closing of the six months' colleges affords an opportunity for the profession in general and the veterinary organizations over the land to review the work done by those institutions. We are sure that in some the course during the past year has not been all that it should have been, and that we are suffering to-day from too many schools which rob those whose facilities and equipments are good of that support to which they are entitled. It is a loss to the student fraternity, to the profession, and to future clientages who must suffer serious losses while an imperfect education is being completed. The time has come when post-graduate courses must be given in the colleges, the State Veterinary Boards must draw their lines closer and closer, and the Associations must stand firm in the demand for better work by all of our colleges.

---

THE JOURNAL regrets to announce its inability to make satisfactory arrangements to place our Association Proceedings Department under the direction of Dr. John J. Repp, as previously announced. All records of society proceedings and notices of meetings will be sent as usual direct to the JOURNAL office. We also wish to state that this department in the issues for 1903 were directed from the JOURNAL office.

